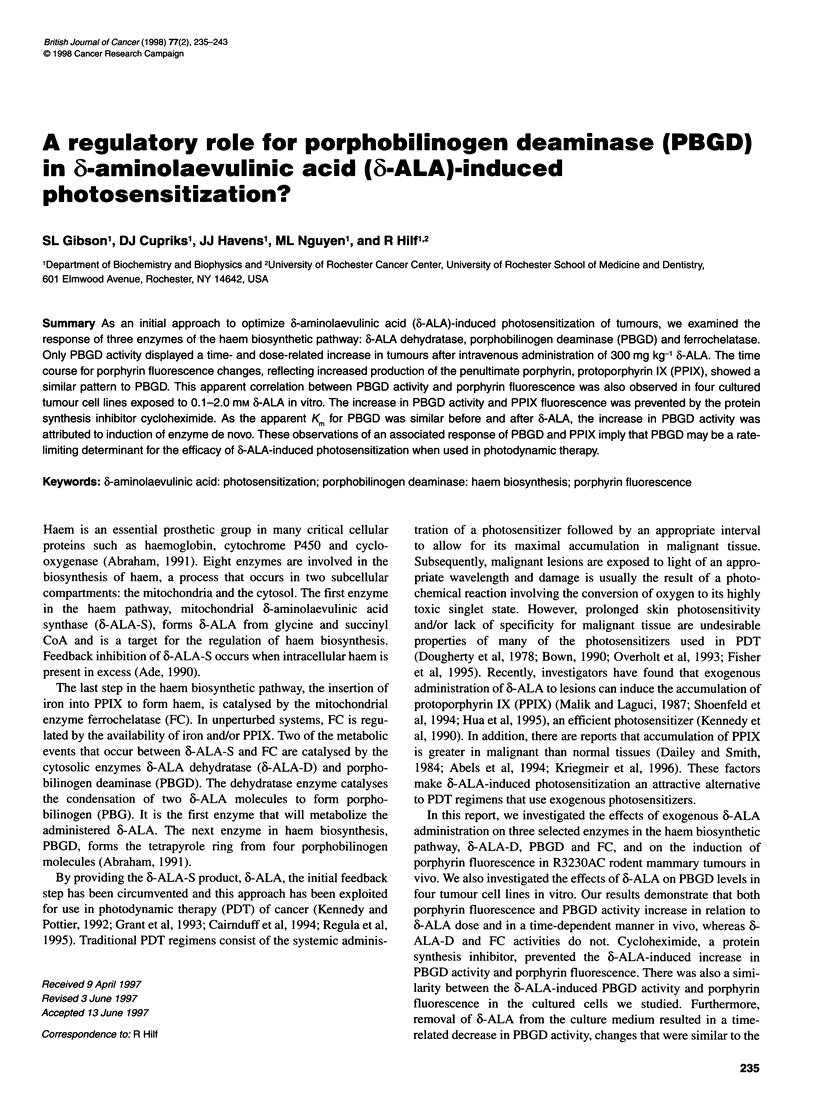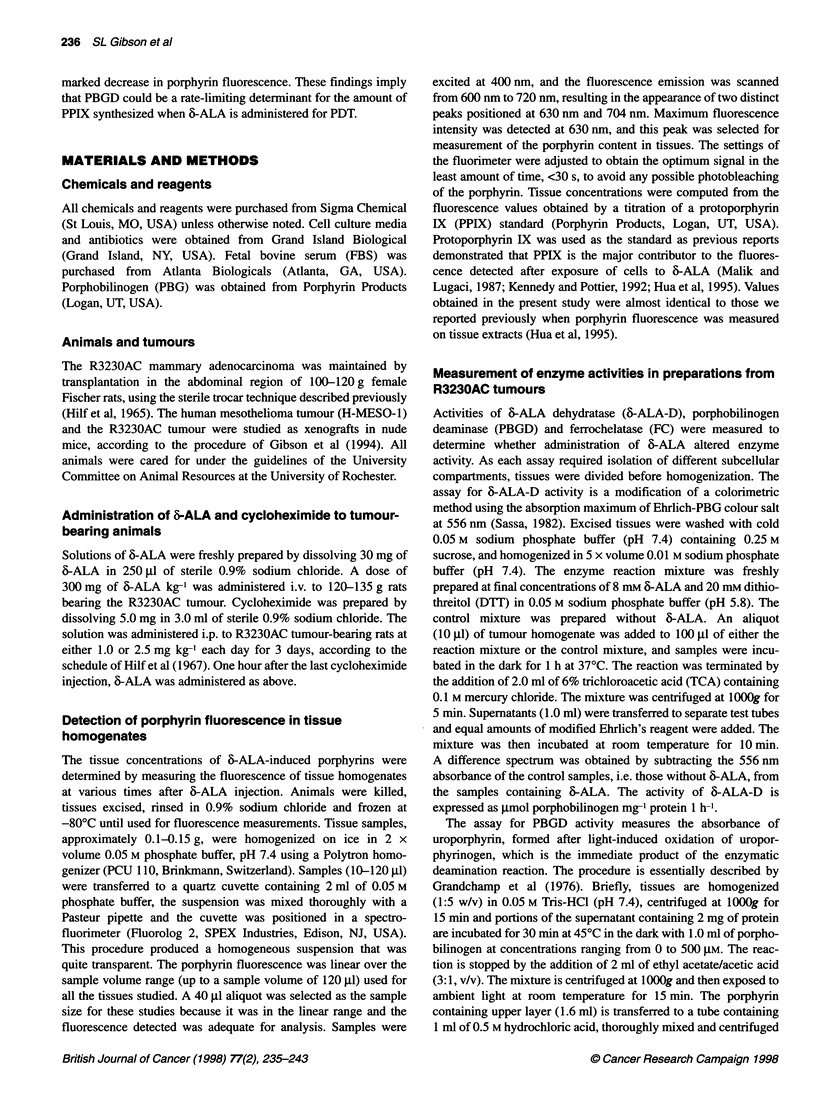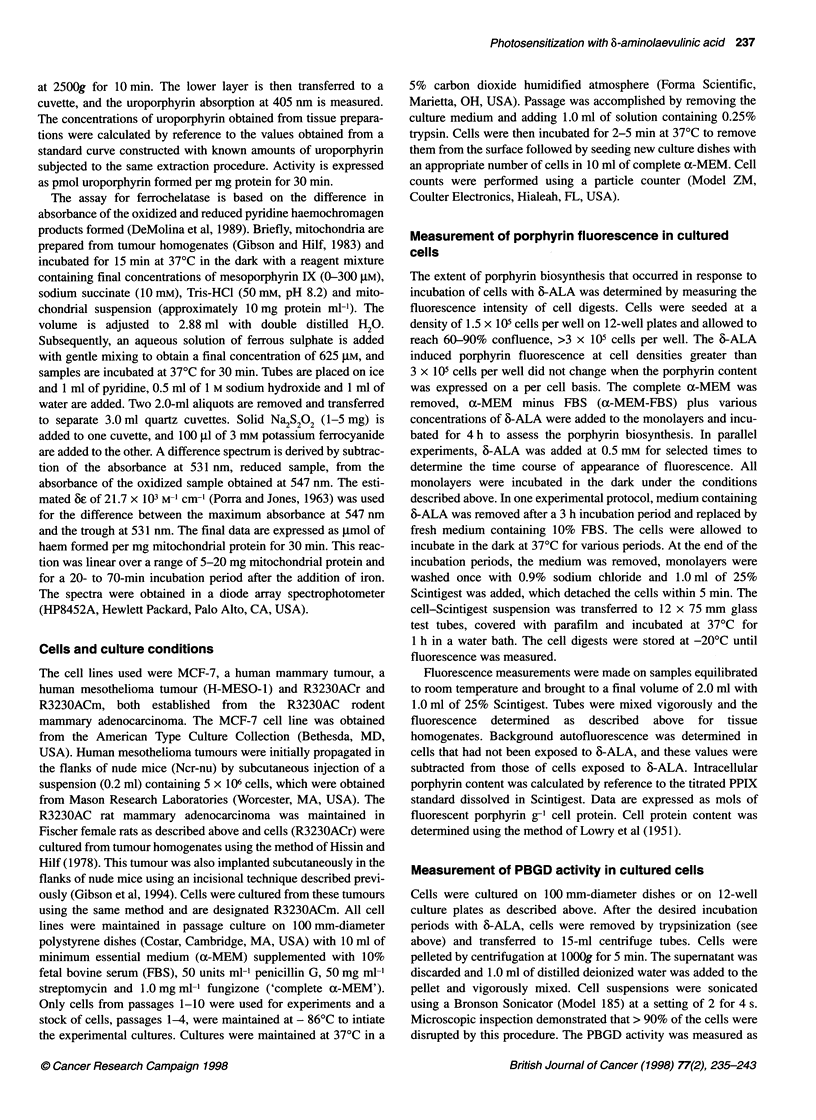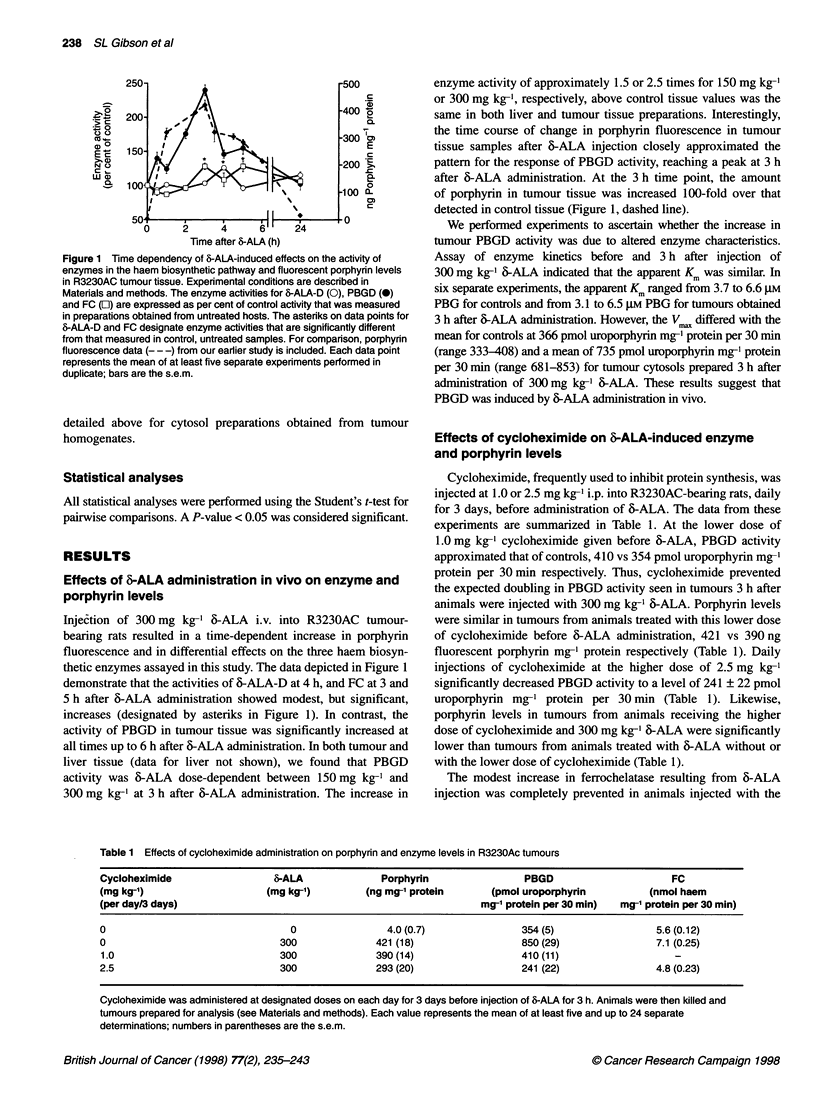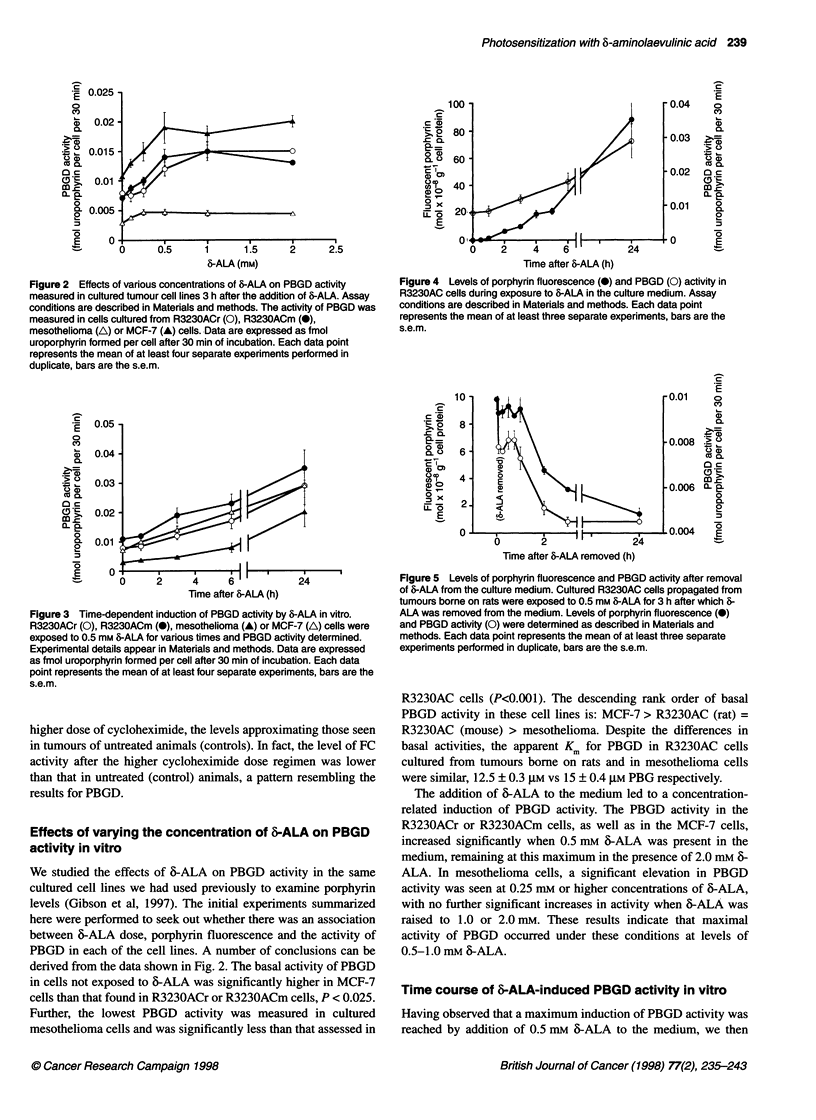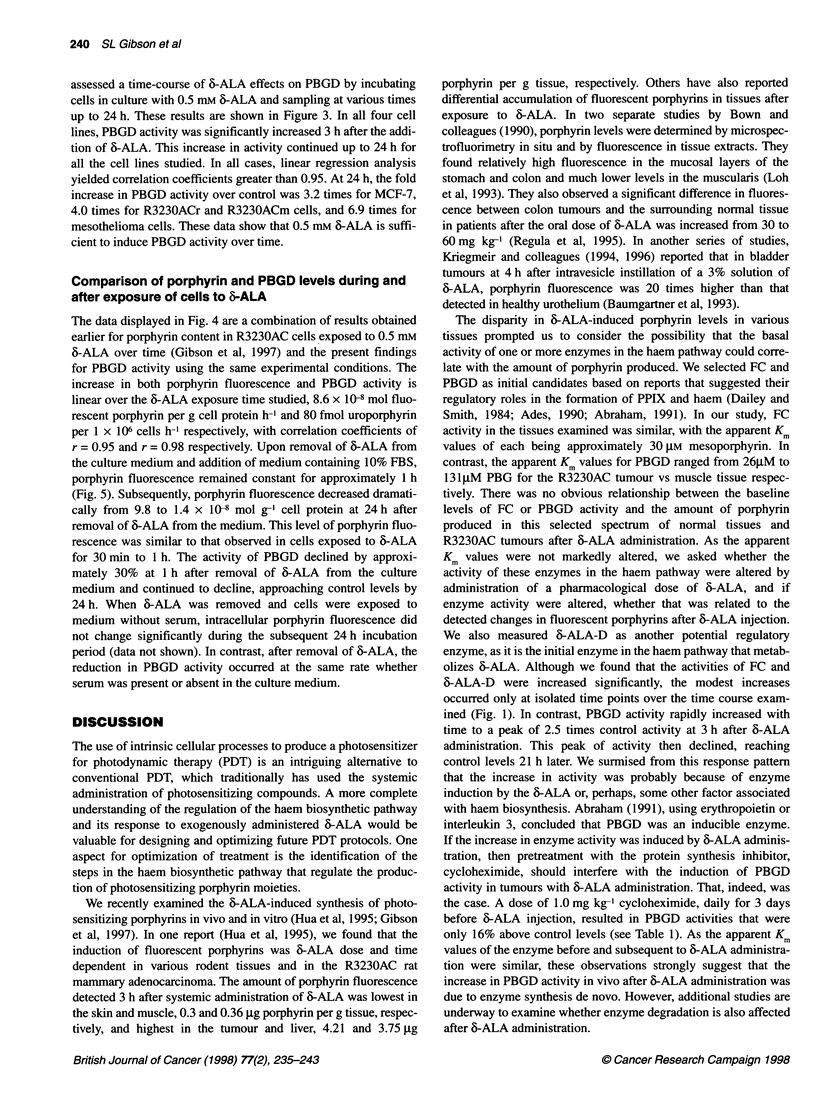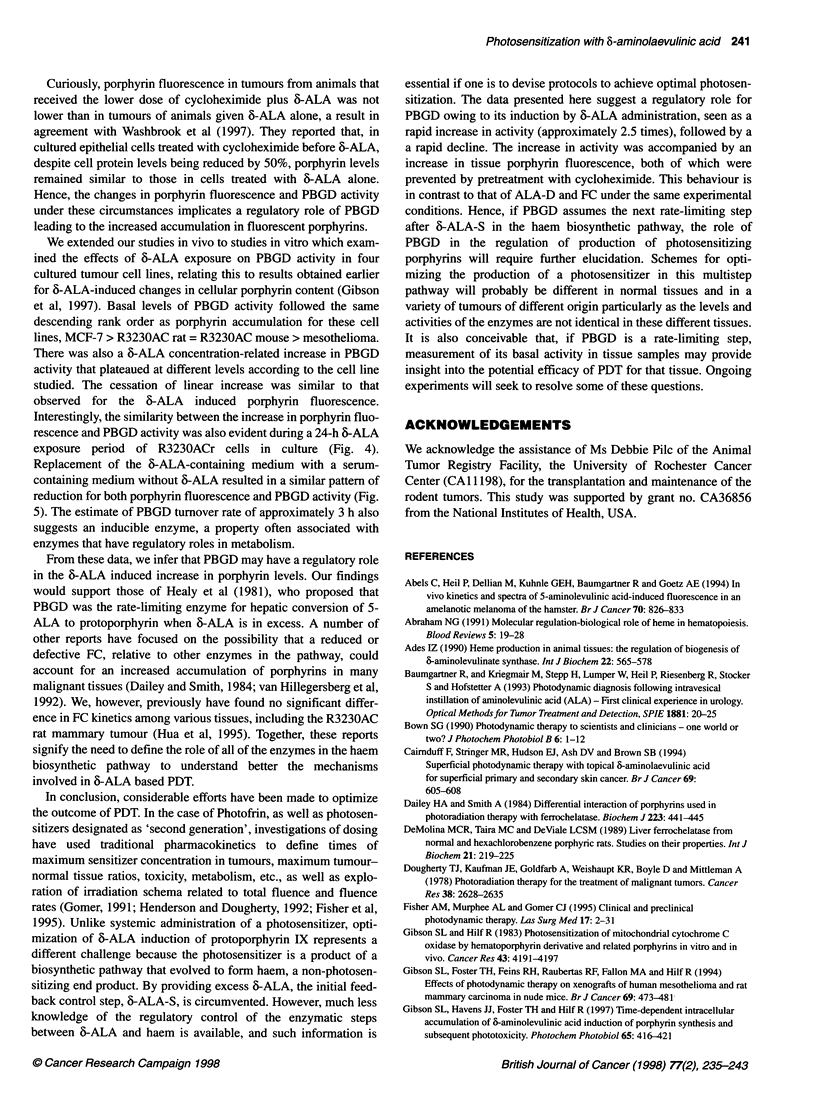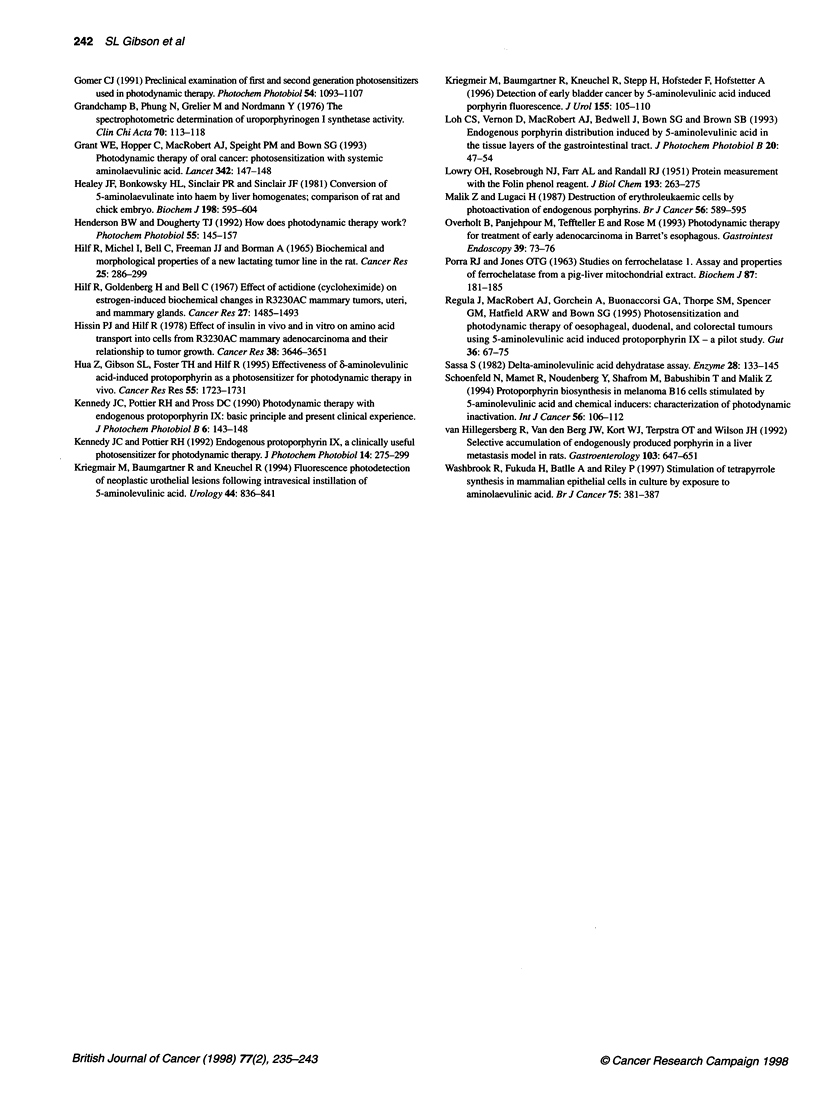# A regulatory role for porphobilinogen deaminase (PBGD) in delta-aminolaevulinic acid (delta-ALA)-induced photosensitization?

**DOI:** 10.1038/bjc.1998.39

**Published:** 1998

**Authors:** S. L. Gibson, D. J. Cupriks, J. J. Havens, M. L. Nguyen, R. Hilf

**Affiliations:** Department of Biochemistry and Biophysics, University of Rochester School of Medicine and Dentistry, NY 14642, USA.

## Abstract

As an initial approach to optimize delta-aminolaevulinic acid (delta-ALA)-induced photosensitization of tumours, we examined the response of three enzymes of the haem biosynthetic pathway: delta-ALA dehydratase, porphobilinogen deaminase (PBGD) and ferrochelatase. Only PBGD activity displayed a time- and dose-related increase in tumours after intravenous administration of 300 mg kg(-1) delta-ALA. The time course for porphyrin fluorescence changes, reflecting increased production of the penultimate porphyrin, protoporphyrin IX (PPIX), showed a similar pattern to PBGD. This apparent correlation between PBGD activity and porphyrin fluorescence was also observed in four cultured tumour cell lines exposed to 0.1-2.0 mM delta-ALA in vitro. The increase in PBGD activity and PPIX fluorescence was prevented by the protein synthesis inhibitor cycloheximide. As the apparent Km for PBGD was similar before and after delta-ALA, the increase in PBGD activity was attributed to induction of enzyme de novo. These observations of an associated response of PBGD and PPIX imply that PBGD may be a rate-limiting determinant for the efficacy of delta-ALA-induced photosensitization when used in photodynamic therapy.


British Journal of Cancer (1998) 77(2), 235–243
© 1998 Cancer Research Campaign

### A regulatory role for porphobilinogen deaminase (PBGD) in δ-aminolaevulinic acid (δ-ALA)-induced photosensitization?

**SL Gibson[1], DJ Cupriks[1], JJ Havens[1], ML Nguyen[1], and R Hilf[1,2]**

[1]Department of Biochemistry and Biophysics and [2]University of Rochester Cancer Center, University of Rochester School of Medicine and Dentistry, 601 Elmwood Avenue, Rochester, NY 14642, USA

**Summary** As an initial approach to optimize δ-aminolaevulinic acid (δ-ALA)-induced photosensitization of tumours, we examined the response of three enzymes of the haem biosynthetic pathway: δ-ALA dehydratase, porphobilinogen deaminase (PBGD) and ferrochelatase. Only PBGD activity displayed a time- and dose-related increase in tumours after intravenous administration of 300 mg $kg^{-1}$ δ-ALA. The time course for porphyrin fluorescence changes, reflecting increased production of the penultimate porphyrin, protoporphyrin IX (PPIX), showed a similar pattern to PBGD. This apparent correlation between PBGD activity and porphyrin fluorescence was also observed in four cultured tumour cell lines exposed to 0.1–2.0 mM δ-ALA in vitro. The increase in PBGD activity and PPIX fluorescence was prevented by the protein synthesis inhibitor cycloheximide. As the apparent $K_m$ for PBGD was similar before and after δ-ALA, the increase in PBGD activity was attributed to induction of enzyme de novo. These observations of an associated response of PBGD and PPIX imply that PBGD may be a rate-limiting determinant for the efficacy of δ-ALA-induced photosensitization when used in photodynamic therapy.

**Keywords:** δ-aminolaevulinic acid: photosensitization; porphobilinogen deaminase: haem biosynthesis; porphyrin fluorescence

Haem is an essential prosthetic group in many critical cellular proteins such as haemoglobin, cytochrome P450 and cyclo-oxygenase (Abraham, 1991). Eight enzymes are involved in the biosynthesis of haem, a process that occurs in two subcellular compartments: the mitochondria and the cytosol. The first enzyme in the haem pathway, mitochondrial δ-aminolaevulinic acid synthase (δ-ALA-S), forms δ-ALA from glycine and succinyl CoA and is a target for the regulation of haem biosynthesis. Feedback inhibition of δ-ALA-S occurs when intracellular haem is present in excess (Ade, 1990).

The last step in the haem biosynthetic pathway, the insertion of iron into PPIX to form haem, is catalysed by the mitochondrial enzyme ferrochelatase (FC). In unperturbed systems, FC is regulated by the availability of iron and/or PPIX. Two of the metabolic events that occur between δ-ALA-S and FC are catalysed by the cytosolic enzymes δ-ALA dehydratase (δ-ALA-D) and porphobilinogen deaminase (PBGD). The dehydratase enzyme catalyses the condensation of two δ-ALA molecules to form porphobilinogen (PBG). It is the first enzyme that will metabolize the administered δ-ALA. The next enzyme in haem biosynthesis, PBGD, forms the tetrapyrole ring from four porphobilinogen molecules (Abraham, 1991).

By providing the δ-ALA-S product, δ-ALA, the initial feedback step has been circumvented and this approach has been exploited for use in photodynamic therapy (PDT) of cancer (Kennedy and Pottier, 1992; Grant et al, 1993; Cairnduff et al, 1994; Regula et al, 1995). Traditional PDT regimens consist of the systemic administration of a photosensitizer followed by an appropriate interval to allow for its maximal accumulation in malignant tissue. Subsequently, malignant lesions are exposed to light of an appropriate wavelength and damage is usually the result of a photochemical reaction involving the conversion of oxygen to its highly toxic singlet state. However, prolonged skin photosensitivity and/or lack of specificity for malignant tissue are undesirable properties of many of the photosensitizers used in PDT (Dougherty et al, 1978; Bown, 1990; Overholt et al, 1993; Fisher et al, 1995). Recently, investigators have found that exogenous administration of δ-ALA to lesions can induce the accumulation of protoporphyrin IX (PPIX) (Malik and Laguci, 1987; Shoenfeld et al, 1994; Hua et al, 1995), an efficient photosensitizer (Kennedy et al, 1990). In addition, there are reports that accumulation of PPIX is greater in malignant than normal tissues (Dailey and Smith, 1984; Abels et al, 1994; Kriegmeir et al, 1996). These factors make δ-ALA-induced photosensitization an attractive alternative to PDT regimens that use exogenous photosensitizers.

In this report, we investigated the effects of exogenous δ-ALA administration on three selected enzymes in the haem biosynthetic pathway, δ-ALA-D, PBGD and FC, and on the induction of porphyrin fluorescence in R3230AC rodent mammary tumours in vivo. We also investigated the effects of δ-ALA on PBGD levels in four tumour cell lines in vitro. Our results demonstrate that both porphyrin fluorescence and PBGD activity increase in relation to δ-ALA dose and in a time-dependent manner in vivo, whereas δ-ALA-D and FC activities do not. Cycloheximide, a protein synthesis inhibitor, prevented the δ-ALA-induced increase in PBGD activity and porphyrin fluorescence. There was also a similarity between the δ-ALA-induced PBGD activity and porphyrin fluorescence in the cultured cells we studied. Furthermore, removal of δ-ALA from the culture medium resulted in a time-related decrease in PBGD activity, changes that were similar to the

*Received 9 April 1997*
*Revised 3 June 1997*
*Accepted 13 June 1997*

*Correspondence to:* R Hilf marked decrease in porphyrin fluorescence. These findings imply that PBGD could be a rate-limiting determinant for the amount of PPIX synthesized when δ-ALA is administered for PDT.

### MATERIALS AND METHODS

### Chemicals and reagents

All chemicals and reagents were purchased from Sigma Chemical (St Louis, MO, USA) unless otherwise noted. Cell culture media and antibiotics were obtained from Grand Island Biological (Grand Island, NY, USA). Fetal bovine serum (FBS) was purchased from Atlanta Biologicals (Atlanta, GA, USA). Porphobilinogen (PBG) was obtained from Porphyrin Products (Logan, UT, USA).

### Animals and tumours

The R3230AC mammary adenocarcinoma was maintained by transplantation in the abdominal region of 100–120 g female Fischer rats, using the sterile trocar technique described previously (Hilf et al, 1965). The human mesothelioma tumour (H-MESO-1) and the R3230AC tumour were studied as xenografts in nude mice, according to the procedure of Gibson et al (1994). All animals were cared for under the guidelines of the University Committee on Animal Resources at the University of Rochester.

### Administration of δ-ALA and cycloheximide to tumour-bearing animals

Solutions of δ-ALA were freshly prepared by dissolving 30 mg of δ-ALA in 250 μl of sterile 0.9% sodium chloride. A dose of 300 mg of δ-ALA $kg^{-1}$ was administered i.v. to 120–135 g rats bearing the R3230AC tumour. Cycloheximide was prepared by dissolving 5.0 mg in 3.0 ml of sterile 0.9% sodium chloride. The solution was administered i.p. to R3230AC tumour-bearing rats at either 1.0 or 2.5 mg $kg^{-1}$ each day for 3 days, according to the schedule of Hilf et al (1967). One hour after the last cycloheximide injection, δ-ALA was administered as above.

### Detection of porphyrin fluorescence in tissue homogenates

The tissue concentrations of δ-ALA-induced porphyrins were determined by measuring the fluorescence of tissue homogenates at various times after δ-ALA injection. Animals were killed, tissues excised, rinsed in 0.9% sodium chloride and frozen at −80°C until used for fluorescence measurements. Tissue samples, approximately 0.1–0.15 g, were homogenized on ice in 2 × volume 0.05 M phosphate buffer, pH 7.4 using a Polytron homogenizer (PCU 110, Brinkmann, Switzerland). Samples (10–120 μl) were transferred to a quartz cuvette containing 2 ml of 0.05 M phosphate buffer, the suspension was mixed thoroughly with a Pasteur pipette and the cuvette was positioned in a spectrofluorimeter (Fluorolog 2, SPEX Industries, Edison, NJ, USA). This procedure produced a homogeneous suspension that was quite transparent. The porphyrin fluorescence was linear over the sample volume range (up to a sample volume of 120 μl) used for all the tissues studied. A 40 μl aliquot was selected as the sample size for these studies because it was in the linear range and the fluorescence detected was adequate for analysis. Samples were excited at 400 nm, and the fluorescence emission was scanned from 600 nm to 720 nm, resulting in the appearance of two distinct peaks positioned at 630 nm and 704 nm. Maximum fluorescence intensity was detected at 630 nm, and this peak was selected for measurement of the porphyrin content in tissues. The settings of the fluorimeter were adjusted to obtain the optimum signal in the least amount of time, <30 s, to avoid any possible photobleaching of the porphyrin. Tissue concentrations were computed from the fluorescence values obtained by a titration of a protoporphyrin IX (PPIX) standard (Porphyrin Products, Logan, UT, USA). Protoporphyrin IX was used as the standard as previous reports demonstrated that PPIX is the major contributor to the fluorescence detected after exposure of cells to δ-ALA (Malik and Lugaci, 1987; Kennedy and Pottier, 1992; Hua et al, 1995). Values obtained in the present study were almost identical to those we reported previously when porphyrin fluorescence was measured on tissue extracts (Hua et al, 1995).

### Measurement of enzyme activities in preparations from R3230AC tumours

Activities of δ-ALA dehydratase (δ-ALA-D), porphobilinogen deaminase (PBGD) and ferrochelatase (FC) were measured to determine whether administration of δ-ALA altered enzyme activity. As each assay required isolation of different subcellular compartments, tissues were divided before homogenization. The assay for δ-ALA-D activity is a modification of a colorimetric method using the absorption maximum of Ehrlich-PBG colour salt at 556 nm (Sassa, 1982). Excised tissues were washed with cold 0.05 M sodium phosphate buffer (pH 7.4) containing 0.25 M sucrose, and homogenized in 5 × volume 0.01 M sodium phosphate buffer (pH 7.4). The enzyme reaction mixture was freshly prepared at final concentrations of 8 mM δ-ALA and 20 mM dithiothreitol (DTT) in 0.05 M sodium phosphate buffer (pH 5.8). The control mixture was prepared without δ-ALA. An aliquot (10 μl) of tumour homogenate was added to 100 μl of either the reaction mixture or the control mixture, and samples were incubated in the dark for 1 h at 37°C. The reaction was terminated by the addition of 2.0 ml of 6% trichloroacetic acid (TCA) containing 0.1 M mercury chloride. The mixture was centrifuged at 1000*g* for 5 min. Supernatants (1.0 ml) were transferred to separate test tubes and equal amounts of modified Ehrlich's reagent were added. The mixture was then incubated at room temperature for 10 min. A difference spectrum was obtained by subtracting the 556 nm absorbance of the control samples, i.e. those without δ-ALA, from the samples containing δ-ALA. The activity of δ-ALA-D is expressed as μmol porphobilinogen $mg^{-1}$ protein 1 $h^{-1}$.

The assay for PBGD activity measures the absorbance of uroporphyrin, formed after light-induced oxidation of uroporphyrinogen, which is the immediate product of the enzymatic deamination reaction. The procedure is essentially described by Grandchamp et al (1976). Briefly, tissues are homogenized (1:5 w/v) in 0.05 M Tris-HCl (pH 7.4), centrifuged at 1000*g* for 15 min and portions of the supernatant containing 2 mg of protein are incubated for 30 min at 45°C in the dark with 1.0 ml of porphobilinogen at concentrations ranging from 0 to 500 μM. The reaction is stopped by the addition of 2 ml of ethyl acetate/acetic acid (3:1, v/v). The mixture is centrifuged at 1000*g* and then exposed to ambient light at room temperature for 15 min. The porphyrin containing upper layer (1.6 ml) is transferred to a tube containing 1 ml of 0.5 M hydrochloric acid, thoroughly mixed and centrifuged

© Cancer Research Campaign 1998

at 2500*g* for 10 min. The lower layer is then transferred to a cuvette, and the uroporphyrin absorption at 405 nm is measured. The concentrations of uroporphyrin obtained from tissue preparations were calculated by reference to the values obtained from a standard curve constructed with known amounts of uroporphyrin subjected to the same extraction procedure. Activity is expressed as pmol uroporphyrin formed per mg protein for 30 min.

The assay for ferrochelatase is based on the difference in absorbance of the oxidized and reduced pyridine haemochromagen products formed (DeMolina et al, 1989). Briefly, mitochondria are prepared from tumour homogenates (Gibson and Hilf, 1983) and incubated for 15 min at 37°C in the dark with a reagent mixture containing final concentrations of mesoporphyrin IX (0–300 μM), sodium succinate (10 mM), Tris-HCl (50 mM, pH 8.2) and mitochondrial suspension (approximately 10 mg protein $ml^{-1}$). The volume is adjusted to 2.88 ml with double distilled $H_2O$. Subsequently, an aqueous solution of ferrous sulphate is added with gentle mixing to obtain a final concentration of 625 μM, and samples are incubated at 37°C for 30 min. Tubes are placed on ice and 1 ml of pyridine, 0.5 ml of 1 M sodium hydroxide and 1 ml of water are added. Two 2.0-ml aliquots are removed and transferred to separate 3.0 ml quartz cuvettes. Solid $Na_2S_2O_2$ (1–5 mg) is added to one cuvette, and 100 μl of 3 mM potassium ferrocyanide are added to the other. A difference spectrum is derived by subtraction of the absorbance at 531 nm, reduced sample, from the absorbance of the oxidized sample obtained at 547 nm. The estimated $\delta\varepsilon$ of $21.7 \times 10^3$ $M^{-1}$ $cm^{-1}$ (Porra and Jones, 1963) was used for the difference between the maximum absorbance at 547 nm and the trough at 531 nm. The final data are expressed as μmol of haem formed per mg mitochondrial protein for 30 min. This reaction was linear over a range of 5–20 mg mitochondrial protein and for a 20- to 70-min incubation period after the addition of iron. The spectra were obtained in a diode array spectrophotometer (HP8452A, Hewlett Packard, Palo Alto, CA, USA).

### Cells and culture conditions

The cell lines used were MCF-7, a human mammary tumour, a human mesothelioma tumour (H-MESO-1) and R3230ACr and R3230ACm, both established from the R3230AC rodent mammary adenocarcinoma. The MCF-7 cell line was obtained from the American Type Culture Collection (Bethesda, MD, USA). Human mesothelioma tumours were initially propagated in the flanks of nude mice (Ncr-nu) by subcutaneous injection of a suspension (0.2 ml) containing $5 \times 10^6$ cells, which were obtained from Mason Research Laboratories (Worcester, MA, USA). The R3230AC rat mammary adenocarcinoma was maintained in Fischer female rats as described above and cells (R3230ACr) were cultured from tumour homogenates using the method of Hissin and Hilf (1978). This tumour was also implanted subcutaneously in the flanks of nude mice using an incisional technique described previously (Gibson et al, 1994). Cells were cultured from these tumours using the same method and are designated R3230ACm. All cell lines were maintained in passage culture on 100 mm-diameter polystyrene dishes (Costar, Cambridge, MA, USA) with 10 ml of minimum essential medium (α-MEM) supplemented with 10% fetal bovine serum (FBS), 50 units $ml^{-1}$ penicillin G, 50 mg $ml^{-1}$ streptomycin and 1.0 mg $ml^{-1}$ fungizone ('complete α-MEM'). Only cells from passages 1–10 were used for experiments and a stock of cells, passages 1–4, were maintained at – 86°C to intiate the experimental cultures. Cultures were maintained at 37°C in a 5% carbon dioxide humidified atmosphere (Forma Scientific, Marietta, OH, USA). Passage was accomplished by removing the culture medium and adding 1.0 ml of solution containing 0.25% trypsin. Cells were then incubated for 2–5 min at 37°C to remove them from the surface followed by seeding new culture dishes with an appropriate number of cells in 10 ml of complete α-MEM. Cell counts were performed using a particle counter (Model ZM, Coulter Electronics, Hialeah, FL, USA).

### Measurement of porphyrin fluorescence in cultured cells

The extent of porphyrin biosynthesis that occurred in response to incubation of cells with δ-ALA was determined by measuring the fluorescence intensity of cell digests. Cells were seeded at a density of $1.5 \times 10^5$ cells per well on 12-well plates and allowed to reach 60–90% confluence, $>3 \times 10^5$ cells per well. The δ-ALA induced porphyrin fluorescence at cell densities greater than $3 \times 10^5$ cells per well did not change when the porphyrin content was expressed on a per cell basis. The complete α-MEM was removed, α-MEM minus FBS (α-MEM-FBS) plus various concentrations of δ-ALA were added to the monolayers and incubated for 4 h to assess the porphyrin biosynthesis. In parallel experiments, δ-ALA was added at 0.5 mM for selected times to determine the time course of appearance of fluorescence. All monolayers were incubated in the dark under the conditions described above. In one experimental protocol, medium containing δ-ALA was removed after a 3 h incubation period and replaced by fresh medium containing 10% FBS. The cells were allowed to incubate in the dark at 37°C for various periods. At the end of the incubation periods, the medium was removed, monolayers were washed once with 0.9% sodium chloride and 1.0 ml of 25% Scintigest was added, which detached the cells within 5 min. The cell–Scintigest suspension was transferred to 12 × 75 mm glass test tubes, covered with parafilm and incubated at 37°C for 1 h in a water bath. The cell digests were stored at –20°C until fluorescence was measured.

Fluorescence measurements were made on samples equilibrated to room temperature and brought to a final volume of 2.0 ml with 1.0 ml of 25% Scintigest. Tubes were mixed vigorously and the fluorescence determined as described above for tissue homogenates. Background autofluorescence was determined in cells that had not been exposed to δ-ALA, and these values were subtracted from those of cells exposed to δ-ALA. Intracellular porphyrin content was calculated by reference to the titrated PPIX standard dissolved in Scintigest. Data are expressed as mols of fluorescent porphyrin $g^{-1}$ cell protein. Cell protein content was determined using the method of Lowry et al (1951).

### Measurement of PBGD activity in cultured cells

Cells were cultured on 100 mm-diameter dishes or on 12-well culture plates as described above. After the desired incubation periods with δ-ALA, cells were removed by trypsinization (see above) and transferred to 15-ml centrifuge tubes. Cells were pelleted by centrifugation at 1000*g* for 5 min. The supernatant was discarded and 1.0 ml of distilled deionized water was added to the pellet and vigorously mixed. Cell suspensions were sonicated using a Bronson Sonicator (Model 185) at a setting of 2 for 4 s. Microscopic inspection demonstrated that > 90% of the cells were disrupted by this procedure. The PBGD activity was measured as

**Figure 1** Time dependency of δ-ALA-induced effects on the activity of enzymes in the haem biosynthetic pathway and fluorescent porphyrin levels in R3230AC tumour tissue. Experimental conditions are described in Materials and methods. The enzyme activities for δ-ALA-D (○), PBGD (●) and FC (□) are expressed as per cent of control activity that was measured in preparations obtained from untreated hosts. The asteriks on data points for δ-ALA-D and FC designate enzyme activities that are significantly different from that measured in control, untreated samples. For comparison, porphyrin fluorescence data (– – –) from our earlier study is included. Each data point represents the mean of at least five separate experiments performed in duplicate; bars are the s.e.m.

detailed above for cytosol preparations obtained from tumour homogenates.

### Statistical analyses

All statistical analyses were performed using the Student's *t*-test for pairwise comparisons. A *P*-value < 0.05 was considered significant.

### RESULTS

### Effects of δ-ALA administration in vivo on enzyme and porphyrin levels

Injection of 300 mg kg$^{-1}$ δ-ALA i.v. into R3230AC tumour-bearing rats resulted in a time-dependent increase in porphyrin fluorescence and in differential effects on the three haem biosynthetic enzymes assayed in this study. The data depicted in Figure 1 demonstrate that the activities of δ-ALA-D at 4 h, and FC at 3 and 5 h after δ-ALA administration showed modest, but significant, increases (designated by asteriks in Figure 1). In contrast, the activity of PBGD in tumour tissue was significantly increased at all times up to 6 h after δ-ALA administration. In both tumour and liver tissue (data for liver not shown), we found that PBGD activity was δ-ALA dose-dependent between 150 mg kg$^{-1}$ and 300 mg kg$^{-1}$ at 3 h after δ-ALA administration. The increase in enzyme activity of approximately 1.5 or 2.5 times for 150 mg kg$^{-1}$ or 300 mg kg$^{-1}$, respectively, above control tissue values was the same in both liver and tumour tissue preparations. Interestingly, the time course of change in porphyrin fluorescence in tumour tissue samples after δ-ALA injection closely approximated the pattern for the response of PBGD activity, reaching a peak at 3 h after δ-ALA administration. At the 3 h time point, the amount of porphyrin in tumour tissue was increased 100-fold over that detected in control tissue (Figure 1, dashed line).

We performed experiments to ascertain whether the increase in tumour PBGD activity was due to altered enzyme characteristics. Assay of enzyme kinetics before and 3 h after injection of 300 mg kg$^{-1}$ δ-ALA indicated that the apparent $K_m$ was similar. In six separate experiments, the apparent $K_m$ ranged from 3.7 to 6.6 μM PBG for controls and from 3.1 to 6.5 μM PBG for tumours obtained 3 h after δ-ALA administration. However, the $V_{max}$ differed with the mean for controls at 366 pmol uroporphyrin mg$^{-1}$ protein per 30 min (range 333–408) and a mean of 735 pmol uroporphyrin mg$^{-1}$ protein per 30 min (range 681–853) for tumour cytosols prepared 3 h after administration of 300 mg kg$^{-1}$ δ-ALA. These results suggest that PBGD was induced by δ-ALA administration in vivo.

### Effects of cycloheximide on δ-ALA-induced enzyme and porphyrin levels

Cycloheximide, frequently used to inhibit protein synthesis, was injected at 1.0 or 2.5 mg kg$^{-1}$ i.p. into R3230AC-bearing rats, daily for 3 days, before administration of δ-ALA. The data from these experiments are summarized in Table 1. At the lower dose of 1.0 mg kg$^{-1}$ cycloheximide given before δ-ALA, PBGD activity approximated that of controls, 410 vs 354 pmol uroporphyrin mg$^{-1}$ protein per 30 min respectively. Thus, cycloheximide prevented the expected doubling in PBGD activity seen in tumours 3 h after animals were injected with 300 mg kg$^{-1}$ δ-ALA. Porphyrin levels were similar in tumours from animals treated with this lower dose of cycloheximide before δ-ALA administration, 421 vs 390 ng fluorescent porphyrin mg$^{-1}$ protein respectively (Table 1). Daily injections of cycloheximide at the higher dose of 2.5 mg kg$^{-1}$ significantly decreased PBGD activity to a level of 241 ± 22 pmol uroporphyrin mg$^{-1}$ protein per 30 min (Table 1). Likewise, porphyrin levels in tumours from animals receiving the higher dose of cycloheximide and 300 mg kg$^{-1}$ δ-ALA were significantly lower than tumours from animals treated with δ-ALA without or with the lower dose of cycloheximide (Table 1).

The modest increase in ferrochelatase resulting from δ-ALA injection was completely prevented in animals injected with the

**Table 1** Effects of cycloheximide administration on porphyrin and enzyme levels in R3230Ac tumours

| Cycloheximide (mg kg$^{-1}$) (per day/3 days) | δ-ALA (mg kg$^{-1}$) | Porphyrin (ng mg$^{-1}$ protein | PBGD (pmol uroporphyrin mg$^{-1}$ protein per 30 min) | FC (nmol haem mg$^{-1}$ protein per 30 min) |
|---|---|---|---|---|
| 0 | 0 | 4.0 (0.7) | 354 (5) | 5.6 (0.12) |
| 0 | 300 | 421 (18) | 850 (29) | 7.1 (0.25) |
| 1.0 | 300 | 390 (14) | 410 (11) | – |
| 2.5 | 300 | 293 (20) | 241 (22) | 4.8 (0.23) |

Cycloheximide was administered at designated doses on each day for 3 days before injection of δ-ALA for 3 h. Animals were then killed and tumours prepared for analysis (see Materials and methods). Each value represents the mean of at least five and up to 24 separate determinations; numbers in parentheses are the s.e.m.

 © Cancer Research Campaign 1998

**Figure 2** Effects of various concentrations of δ-ALA on PBGD activity measured in cultured tumour cell lines 3 h after the addition of δ-ALA. Assay conditions are described in Materials and methods. The activity of PBGD was measured in cells cultured from R3230ACr (○), R3230ACm (●), mesothelioma (△) or MCF-7 (▲) cells. Data are expressed as fmol uroporphyrin formed per cell after 30 min of incubation. Each data point represents the mean of at least four separate experiments performed in duplicate, bars are the s.e.m.

**Figure 3** Time-dependent induction of PBGD activity by δ-ALA in vitro. R3230ACr (○), R3230ACm (●), mesothelioma (▲) or MCF-7 (△) cells were exposed to 0.5 mM δ-ALA for various times and PBGD activity determined. Experimental details appear in Materials and methods. Data are expressed as fmol uroporphyrin formed per cell after 30 min of incubation. Each data point represents the mean of at least four separate experiments, bars are the s.e.m.

**Figure 4** Levels of porphyrin fluorescence (●) and PBGD (○) activity in R3230AC cells during exposure to δ-ALA in the culture medium. Assay conditions are described in Materials and methods. Each data point represents the mean of at least three separate experiments, bars are the s.e.m.

**Figure 5** Levels of porphyrin fluorescence and PBGD activity after removal of δ-ALA from the culture medium. Cultured R3230AC cells propagated from tumours borne on rats were exposed to 0.5 mM δ-ALA for 3 h after which δ-ALA was removed from the medium. Levels of porphyrin fluorescence (●) and PBGD activity (○) were determined as described in Materials and methods. Each data point represents the mean of at least three separate experiments performed in duplicate, bars are the s.e.m.

higher dose of cycloheximide, the levels approximating those seen in tumours of untreated animals (controls). In fact, the level of FC activity after the higher cycloheximide dose regimen was lower than that in untreated (control) animals, a pattern resembling the results for PBGD.

### Effects of varying the concentration of δ-ALA on PBGD activity in vitro

We studied the effects of δ-ALA on PBGD activity in the same cultured cell lines we had used previously to examine porphyrin levels (Gibson et al, 1997). The initial experiments summarized here were performed to seek out whether there was an association between δ-ALA dose, porphyrin fluorescence and the activity of PBGD in each of the cell lines. A number of conclusions can be derived from the data shown in Fig. 2. The basal activity of PBGD in cells not exposed to δ-ALA was significantly higher in MCF-7 cells than that found in R3230ACr or R3230ACm cells, $P < 0.025$. Further, the lowest PBGD activity was measured in cultured mesothelioma cells and was significantly less than that assessed in R3230AC cells ($P<0.001$). The descending rank order of basal PBGD activity in these cell lines is: MCF-7 > R3230AC (rat) = R3230AC (mouse) > mesothelioma. Despite the differences in basal activities, the apparent $K_m$ for PBGD in R3230AC cells cultured from tumours borne on rats and in mesothelioma cells were similar, 12.5 ± 0.3 μM vs 15 ± 0.4 μM PBG respectively.

The addition of δ-ALA to the medium led to a concentration-related induction of PBGD activity. The PBGD activity in the R3230ACr or R3230ACm cells, as well as in the MCF-7 cells, increased significantly when 0.5 mM δ-ALA was present in the medium, remaining at this maximum in the presence of 2.0 mM δ-ALA. In mesothelioma cells, a significant elevation in PBGD activity was seen at 0.25 mM or higher concentrations of δ-ALA, with no further significant increases in activity when δ-ALA was raised to 1.0 or 2.0 mM. These results indicate that maximal activity of PBGD occurred under these conditions at levels of 0.5–1.0 mM δ-ALA.

### Time course of δ-ALA-induced PBGD activity in vitro

Having observed that a maximum induction of PBGD activity was reached by addition of 0.5 mM δ-ALA to the medium, we then assessed a time-course of δ-ALA effects on PBGD by incubating cells in culture with 0.5 mM δ-ALA and sampling at various times up to 24 h. These results are shown in Figure 3. In all four cell lines, PBGD activity was significantly increased 3 h after the addition of δ-ALA. This increase in activity continued up to 24 h for all the cell lines studied. In all cases, linear regression analysis yielded correlation coefficients greater than 0.95. At 24 h, the fold increase in PBGD activity over control was 3.2 times for MCF-7, 4.0 times for R3230ACr and R3230ACm cells, and 6.9 times for mesothelioma cells. These data show that 0.5 mM δ-ALA is sufficient to induce PBGD activity over time.

### Comparison of porphyrin and PBGD levels during and after exposure of cells to δ-ALA

The data displayed in Fig. 4 are a combination of results obtained earlier for porphyrin content in R3230AC cells exposed to 0.5 mM δ-ALA over time (Gibson et al, 1997) and the present findings for PBGD activity using the same experimental conditions. The increase in both porphyrin fluorescence and PBGD activity is linear over the δ-ALA exposure time studied, $8.6 \times 10^{-8}$ mol fluorescent porphyrin per g cell protein $h^{-1}$ and 80 fmol uroporphyrin per $1 \times 10^6$ cells $h^{-1}$ respectively, with correlation coefficients of $r = 0.95$ and $r = 0.98$ respectively. Upon removal of δ-ALA from the culture medium and addition of medium containing 10% FBS, porphyrin fluorescence remained constant for approximately 1 h (Fig. 5). Subsequently, porphyrin fluorescence decreased dramatically from 9.8 to $1.4 \times 10^{-8}$ mol $g^{-1}$ cell protein at 24 h after removal of δ-ALA from the medium. This level of porphyrin fluorescence was similar to that observed in cells exposed to δ-ALA for 30 min to 1 h. The activity of PBGD declined by approximately 30% at 1 h after removal of δ-ALA from the culture medium and continued to decline, approaching control levels by 24 h. When δ-ALA was removed and cells were exposed to medium without serum, intracellular porphyrin fluorescence did not change significantly during the subsequent 24 h incubation period (data not shown). In contrast, after removal of δ-ALA, the reduction in PBGD activity occurred at the same rate whether serum was present or absent in the culture medium.

### DISCUSSION

The use of intrinsic cellular processes to produce a photosensitizer for photodynamic therapy (PDT) is an intriguing alternative to conventional PDT, which traditionally has used the systemic administration of photosensitizing compounds. A more complete understanding of the regulation of the haem biosynthetic pathway and its response to exogenously administered δ-ALA would be valuable for designing and optimizing future PDT protocols. One aspect for optimization of treatment is the identification of the steps in the haem biosynthetic pathway that regulate the production of photosensitizing porphyrin moieties.

We recently examined the δ-ALA-induced synthesis of photosensitizing porphyrins in vivo and in vitro (Hua et al, 1995; Gibson et al, 1997). In one report (Hua et al, 1995), we found that the induction of fluorescent porphyrins was δ-ALA dose and time dependent in various rodent tissues and in the R3230AC rat mammary adenocarcinoma. The amount of porphyrin fluorescence detected 3 h after systemic administration of δ-ALA was lowest in the skin and muscle, 0.3 and 0.36 μg porphyrin per g tissue, respectively, and highest in the tumour and liver, 4.21 and 3.75 μg porphyrin per g tissue, respectively. Others have also reported differential accumulation of fluorescent porphyrins in tissues after exposure to δ-ALA. In two separate studies by Bown and colleagues (1990), porphyrin levels were determined by microspectrofluorimetry in situ and by fluorescence in tissue extracts. They found relatively high fluorescence in the mucosal layers of the stomach and colon and much lower levels in the muscularis (Loh et al, 1993). They also observed a significant difference in fluorescence between colon tumours and the surrounding normal tissue in patients after the oral dose of δ-ALA was increased from 30 to 60 mg $kg^{-1}$ (Regula et al, 1995). In another series of studies, Kriegmeir and colleagues (1994, 1996) reported that in bladder tumours at 4 h after intravesicle instillation of a 3% solution of δ-ALA, porphyrin fluorescence was 20 times higher than that detected in healthy urothelium (Baumgartner et al, 1993).

The disparity in δ-ALA-induced porphyrin levels in various tissues prompted us to consider the possibility that the basal activity of one or more enzymes in the haem pathway could correlate with the amount of porphyrin produced. We selected FC and PBGD as initial candidates based on reports that suggested their regulatory roles in the formation of PPIX and haem (Dailey and Smith, 1984; Ades, 1990; Abraham, 1991). In our study, FC activity in the tissues examined was similar, with the apparent $K_m$ values of each being approximately 30 μM mesoporphyrin. In contrast, the apparent $K_m$ values for PBGD ranged from 26μM to 131μM PBG for the R3230AC tumour vs muscle tissue respectively. There was no obvious relationship between the baseline levels of FC or PBGD activity and the amount of porphyrin produced in this selected spectrum of normal tissues and R3230AC tumours after δ-ALA administration. As the apparent $K_m$ values were not markedly altered, we asked whether the activity of these enzymes in the haem pathway were altered by administration of a pharmacological dose of δ-ALA, and if enzyme activity were altered, whether that was related to the detected changes in fluorescent porphyrins after δ-ALA injection. We also measured δ-ALA-D as another potential regulatory enzyme, as it is the initial enzyme in the haem pathway that metabolizes δ-ALA. Although we found that the activities of FC and δ-ALA-D were increased significantly, the modest increases occurred only at isolated time points over the time course examined (Fig. 1). In contrast, PBGD activity rapidly increased with time to a peak of 2.5 times control activity at 3 h after δ-ALA administration. This peak of activity then declined, reaching control levels 21 h later. We surmised from this response pattern that the increase in activity was probably because of enzyme induction by the δ-ALA or, perhaps, some other factor associated with haem biosynthesis. Abraham (1991), using erythropoietin or interleukin 3, concluded that PBGD was an inducible enzyme. If the increase in enzyme activity was induced by δ-ALA administration, then pretreatment with the protein synthesis inhibitor, cycloheximide, should interfere with the induction of PBGD activity in tumours with δ-ALA administration. That, indeed, was the case. A dose of 1.0 mg $kg^{-1}$ cycloheximide, daily for 3 days before δ-ALA injection, resulted in PBGD activities that were only 16% above control levels (see Table 1). As the apparent $K_m$ values of the enzyme before and subsequent to δ-ALA administration were similar, these observations strongly suggest that the increase in PBGD activity in vivo after δ-ALA administration was due to enzyme synthesis de novo. However, additional studies are underway to examine whether enzyme degradation is also affected after δ-ALA administration.

© Cancer Research Campaign 1998

Curiously, porphyrin fluorescence in tumours from animals that received the lower dose of cycloheximide plus δ-ALA was not lower than in tumours of animals given δ-ALA alone, a result in agreement with Washbrook et al (1997). They reported that, in cultured epithelial cells treated with cycloheximide before δ-ALA, despite cell protein levels being reduced by 50%, porphyrin levels remained similar to those in cells treated with δ-ALA alone. Hence, the changes in porphyrin fluorescence and PBGD activity under these circumstances implicates a regulatory role of PBGD leading to the increased accumulation in fluorescent porphyrins.

We extended our studies in vivo to studies in vitro which examined the effects of δ-ALA exposure on PBGD activity in four cultured tumour cell lines, relating this to results obtained earlier for δ-ALA-induced changes in cellular porphyrin content (Gibson et al, 1997). Basal levels of PBGD activity followed the same descending rank order as porphyrin accumulation for these cell lines, MCF-7 > R3230AC rat = R3230AC mouse > mesothelioma. There was also a δ-ALA concentration-related increase in PBGD activity that plateaued at different levels according to the cell line studied. The cessation of linear increase was similar to that observed for the δ-ALA induced porphyrin fluorescence. Interestingly, the similarity between the increase in porphyrin fluorescence and PBGD activity was also evident during a 24-h δ-ALA exposure period of R3230ACr cells in culture (Fig. 4). Replacement of the δ-ALA-containing medium with a serum-containing medium without δ-ALA resulted in a similar pattern of reduction for both porphyrin fluorescence and PBGD activity (Fig. 5). The estimate of PBGD turnover rate of approximately 3 h also suggests an inducible enzyme, a property often associated with enzymes that have regulatory roles in metabolism.

From these data, we infer that PBGD may have a regulatory role in the δ-ALA induced increase in porphyrin levels. Our findings would support those of Healy et al (1981), who proposed that PBGD was the rate-limiting enzyme for hepatic conversion of 5-ALA to protoporphyrin when δ-ALA is in excess. A number of other reports have focused on the possibility that a reduced or defective FC, relative to other enzymes in the pathway, could account for an increased accumulation of porphyrins in many malignant tissues (Dailey and Smith, 1984; van Hillegersberg et al, 1992). We, however, previously have found no significant difference in FC kinetics among various tissues, including the R3230AC rat mammary tumour (Hua et al, 1995). Together, these reports signify the need to define the role of all of the enzymes in the haem biosynthetic pathway to understand better the mechanisms involved in δ-ALA based PDT.

In conclusion, considerable efforts have been made to optimize the outcome of PDT. In the case of Photofrin, as well as photosensitizers designated as 'second generation', investigations of dosing have used traditional pharmacokinetics to define times of maximum sensitizer concentration in tumours, maximum tumour–normal tissue ratios, toxicity, metabolism, etc., as well as exploration of irradiation schema related to total fluence and fluence rates (Gomer, 1991; Henderson and Dougherty, 1992; Fisher et al, 1995). Unlike systemic administration of a photosensitizer, optimization of δ-ALA induction of protoporphyrin IX represents a different challenge because the photosensitizer is a product of a biosynthetic pathway that evolved to form haem, a non-photosensitizing end product. By providing excess δ-ALA, the initial feedback control step, δ-ALA-S, is circumvented. However, much less knowledge of the regulatory control of the enzymatic steps between δ-ALA and haem is available, and such information is essential if one is to devise protocols to achieve optimal photosensitization. The data presented here suggest a regulatory role for PBGD owing to its induction by δ-ALA administration, seen as a rapid increase in activity (approximately 2.5 times), followed by a a rapid decline. The increase in activity was accompanied by an increase in tissue porphyrin fluorescence, both of which were prevented by pretreatment with cycloheximide. This behaviour is in contrast to that of ALA-D and FC under the same experimental conditions. Hence, if PBGD assumes the next rate-limiting step after δ-ALA-S in the haem biosynthetic pathway, the role of PBGD in the regulation of production of photosensitizing porphyrins will require further elucidation. Schemes for optimizing the production of a photosensitizer in this multistep pathway will probably be different in normal tissues and in a variety of tumours of different origin particularly as the levels and activities of the enzymes are not identical in these different tissues. It is also conceivable that, if PBGD is a rate-limiting step, measurement of its basal activity in tissue samples may provide insight into the potential efficacy of PDT for that tissue. Ongoing experiments will seek to resolve some of these questions.

### ACKNOWLEDGEMENTS

We acknowledge the assistance of Ms Debbie Pilc of the Animal Tumor Registry Facility, the University of Rochester Cancer Center (CA11198), for the transplantation and maintenance of the rodent tumors. This study was supported by grant no. CA36856 from the National Institutes of Health, USA.

### REFERENCES

Abels C, Heil P, Dellian M, Kuhnle GEH, Baumgartner R and Goetz AE (1994) In vivo kinetics and spectra of 5-aminolevulinic acid-induced fluorescence in an amelanotic melanoma of the hamster. *Br J Cancer* **70**: 826–833

Abraham NG (1991) Molecular regulation-biological role of heme in hematopoiesis. *Blood Reviews* **5**: 19–28

Ades IZ (1990) Heme production in animal tissues: the regulation of biogenesis of δ-aminolevulinate synthase. *Int J Biochem* **22**: 565–578

Baumgartner R, and Kriegmair M, Stepp H, Lumper W, Heil P, Riesenberg R, Stocker S and Hofstetter A (1993) Photodynamic diagnosis following intravesical instillation of aminolevulinic acid (ALA) – First clinical experience in urology. *Optical Methods for Tumor Treatment and Detection, SPIE* **1881**: 20–25

Bown SG (1990) Photodynamic therapy to scientists and clinicians – one world or two? *J Photochem Photobiol B* **6**: 1–12

Cairnduff F, Stringer MR, Hudson EJ, Ash DV and Brown SB (1994) Superficial photodynamic therapy with topical δ-aminolaevulinic acid for superficial primary and secondary skin cancer. *Br J Cancer* **69**: 605–608

Dailey HA and Smith A (1984) Differential interaction of porphyrins used in photoradiation therapy with ferrochelatase. *Biochem J* **223**: 441–445

DeMolina MCR, Taira MC and DeViale LCSM (1989) Liver ferrochelatase from normal and hexachlorobenzene porphyric rats. Studies on their properties. *Int J Biochem* **21**: 219–225

Dougherty TJ, Kaufman JE, Goldfarb A, Weishaupt KR, Boyle D and Mittleman A (1978) Photoradiation therapy for the treatment of malignant tumors. *Cancer Res* **38**: 2628–2635

Fisher AM, Murphee AL and Gomer CJ (1995) Clinical and preclinical photodynamic therapy. *Las Surg Med* **17**: 2–31

Gibson SL and Hilf R (1983) Photosensitization of mitochondrial cytochrome C oxidase by hematoporphyrin derivative and related porphyrins in vitro and in vivo. *Cancer Res* **43**: 4191–4197

Gibson SL, Foster TH, Feins RH, Raubertas RF, Fallon MA and Hilf R (1994) Effects of photodynamic therapy on xenografts of human mesothelioma and rat mammary carcinoma in nude mice. *Br J Cancer* **69**: 473–481

Gibson SL, Havens JJ, Foster TH and Hilf R (1997) Time-dependent intracellular accumulation of δ-aminolevulinic acid induction of porphyrin synthesis and subsequent phototoxicity. *Photochem Photobiol* **65**: 416–421

© Cancer Research Campaign 1998

Gomer CJ (1991) Preclinical examination of first and second generation photosensitizers used in photodynamic therapy. *Photochem Photobiol* **54**: 1093–1107

Grandchamp B, Phung N, Grelier M and Nordmann Y (1976) The spectrophotometric determination of uroporphyrinogen I synthetase activity. *Clin Chi Acta* **70**: 113–118

Grant WE, Hopper C, MacRobert AJ, Speight PM and Bown SG (1993) Photodynamic therapy of oral cancer: photosensitization with systemic aminolaevulinic acid. *Lancet* **342**: 147–148

Healey JF, Bonkowsky HL, Sinclair PR and Sinclair JF (1981) Conversion of 5-aminolaevulinate into haem by liver homogenates; comparison of rat and chick embryo. *Biochem J* **198**: 595–604

Henderson BW and Dougherty TJ (1992) How does photodynamic therapy work? *Photochem Photobiol* **55**: 145–157

Hilf R, Michel I, Bell C, Freeman JJ and Borman A (1965) Biochemical and morphological properties of a new lactating tumor line in the rat. *Cancer Res* **25**: 286–299

Hilf R, Goldenberg H and Bell C (1967) Effect of actidione (cycloheximide) on estrogen-induced biochemical changes in R3230AC mammary tumors, uteri, and mammary glands. *Cancer Res* **27**: 1485–1493

Hissin PJ and Hilf R (1978) Effect of insulin in vivo and in vitro on amino acid transport into cells from R3230AC mammary adenocarcinoma and their relationship to tumor growth. *Cancer Res* **38**: 3646–3651

Hua Z, Gibson SL, Foster TH and Hilf R (1995) Effectiveness of δ-aminolevulinic acid-induced protoporphyrin as a photosensitizer for photodynamic therapy in vivo. *Cancer Res* Res **55**: 1723–1731

Kennedy JC, Pottier RH and Pross DC (1990) Photodynamic therapy with endogenous protoporphyrin IX: basic principle and present clinical experience. *J Photochem Photobiol B* **6**: 143–148

Kennedy JC and Pottier RH (1992) Endogenous protoporphyrin IX, a clinically useful photosensitizer for photodynamic therapy. J *Photochem Photobiol* **14**: 275–299

Kriegmair M, Baumgartner R and Kneuchel R (1994) Fluorescence photodetection of neoplastic urothelial lesions following intravesical instillation of 5-aminolevulinic acid. *Urology* **44**: 836–841

Kriegmeir M, Baumgartner R, Kneuchel R, Stepp H, Hofsteder F, Hofstetter A (1996) Detection of early bladder cancer by 5-aminolevulinic acid induced porphyrin fluorescence. *J Urol* **155**: 105–110

Loh CS, Vernon D, MacRobert AJ, Bedwell J, Bown SG and Brown SB (1993) Endogenous porphyrin distribution induced by 5-aminolevulinic acid in the tissue layers of the gastrointestinal tract. *J Photochem Photobiol B* **20**: 47–54

Lowry OH, Rosebrough NJ, Farr AL and Randall RJ (1951) Protein measurement with the Folin phenol reagent. *J Biol Chem* **193**: 263–275

Malik Z and Lugaci H (1987) Destruction of erythroleukaemic cells by photoactivation of endogenous porphyrins. *Br J Cancer* **56**: 589–595

Overholt B, Panjehpour M, Teffteller E and Rose M (1993) Photodynamic therapy for treatment of early adenocarcinoma in Barret's esophagous. *Gastrointest Endoscopy* **39**: 73–76

Porra RJ and Jones OTG (1963) Studies on ferrochelatase 1. Assay and properties of ferrochelatase from a pig-liver mitochondrial extract. *Biochem J* **87**: 181–185

Regula J, MacRobert AJ, Gorchein A, Buonaccorsi GA, Thorpe SM, Spencer GM, Hatfield ARW and Bown SG (1995) Photosensitization and photodynamic therapy of oesophageal, duodenal, and colorectal tumours using 5-aminolevulinic acid induced protoporphyrin IX – a pilot study. *Gut* **36**: 67–75

Sassa S (1982) Delta-aminolevulinic acid dehydratase assay. *Enzyme* **28**: 133–145

Schoenfeld N, Mamet R, Noudenberg Y, Shafrom M, Babushibin T and Malik Z (1994) Protoporphyrin biosynthesis in melanoma B16 cells stimulated by 5-aminolevulinic acid and chemical inducers: characterization of photodynamic inactivation. *Int J Cancer* **56**: 106–112

van Hillegersberg R, Van den Berg JW, Kort WJ, Terpstra OT and Wilson JH (1992) Selective accumulation of endogenously produced porphyrin in a liver metastasis model in rats. *Gastroenterology* **103**: 647–651

Washbrook R, Fukuda H, Batlle A and Riley P (1997) Stimulation of tetrapyrrole synthesis in mammalian epithelial cells in culture by exposure to aminolaevulinic acid. *Br J Cancer* **75**: 381–387

© Cancer Research Campaign 1998